# Molecular Detection of *Histoplasma capsulatum* in Antarctica

**DOI:** 10.3201/eid2810.220046

**Published:** 2022-10

**Authors:** Lucas Machado Moreira, Wieland Meyer, Márcia Chame, Martha Lima Brandão, Adriana Marcos Vivoni, Juana Portugal, Bodo Wanke, Luciana Trilles

**Affiliations:** Oswaldo Cruz Foundation, Rio de Janeiro, Brazil (L.M. Moreira, M. Chame, M.L. Brandão, A.M. Vivoni, J. Portugal, B. Wanke, L. Trilles);; Sydney University, Sydney, New South Wales, Australia (W. Meyer);; Curtin University, Perth, Western Australia, Australia (W. Meyer)

**Keywords:** *Histoplasma*, Antarctic regions, fungi, histoplasmosis, Antarctica

## Abstract

We detected *Histoplasma capsulatum* in soil and penguin excreta in the Antarctic Peninsula by sequencing after performing species-specific PCR, confirming previous observations that this pathogen occurs more broadly than suspected. This finding highlights the need for surveillance of emerging agents of systemic mycoses and their transmission among regions, animals, and humans in Antarctica.

*Histoplasma capsulatum* is a dimorphic fungus of the order Onygenales, which can cause systemic mycosis when inhaled (*1*). The filamentous phase of the fungus usually inhabits environments rich in phosphate and nitrogenous compounds, typically coming primarily from bird or bat droppings. Human intervention and other disturbances to those environments promote dispersion of fungal propagules (spores) in the air, which enables the inhalation of infectious particles (*2*). This pathogen has a wide variety of hosts in addition to humans, and its close relationship with vertebrates suggests that birds and mammals can play a crucial role in the dispersal of the members of this species complex (*3*).

Histoplasmosis occurs worldwide; prevalence varies from low in Europe and Oceania to moderate in Africa and South Asia to high in North America, Central America, and South America. Among areas where it is most prevalent, Latin America is the region with the largest number of cases (*3*).

The genus *Histoplasma* is composed of multiple genetically distinct clades, which differ in geographic distribution, virulence, and progression of pathology (*4*). Kasuga et al. (*4*) evaluated the population genetic diversity of isolates from different countries and continents by using 4 partial protein coding regions and suggested dividing *H. capsulatum* into 7 phylogenetic species (*4*). Those results initiated a whole-genome study to evaluate the species complex, proposing 4 genetically different species: *H. capsulatum* (Panamanian linage), *H. mississippiense* (NAm1), *H. ohiense* (NAm2), and *H. suramericanum* (LAmA) (*5*).

Antarctica is the most isolated and inhospitable continent on the planet. Over the past 2 decades, however, the intensity of human activity has continued to increase, driven by not just explorers but also scientific researchers, station support personnel, fishers, whalers, and, more recently, tourists. These increased human activities have a substantial effect on all life forms in Antarctica, transporting nonindigenous species to the continent and exporting endemic and autochthones species to other continents, including human, animal and plant pathogenic fungi (*6*,*7*). However, pathogenic fungi are rarely explored in the Antarctic setting (*8*), and their effect on visitors to Antarctica and on the human populations in other continents is underinvestigated. This study describes the molecular detection of *H. capsulatum* in soil and penguin excreta in the Antarctic Peninsula.

## The Study

**Figure 1:**
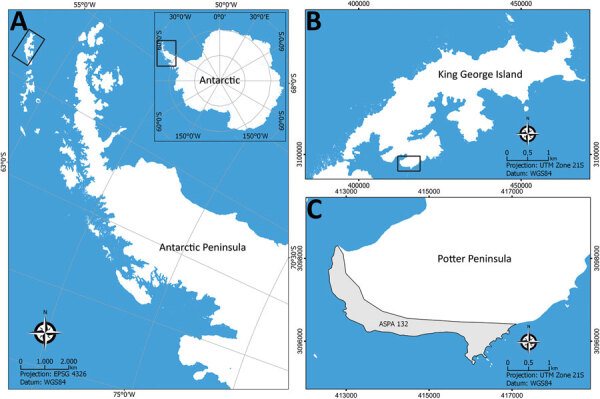
Sampling locations for study of *Histoplasma capsulatum* in Antarctica. A) Location of the Antarctic Peninsula in the Antarctica continent; B) King George Island; C) Potter Peninsula and the Antarctic Specially Protected Area ASPA N°132. Source: SCAR Antarctic Digital Database (https://www.scar.org/resources/antarctic-digital-database).

We collected environmental samples in the Potter Peninsula, an Antarctic Special Protected Area located in King George Island, during the summer of 2020 (Figure 1). In total, we collected 9 samples of penguin excreta, 3 samples of fur seal feces, and 8 samples of superficial soil using sterile material and kept them at 2°C–8°C until analysis.

We extracted DNA from the environmental samples and monitored crossover contamination by including 1 sterile water sample at every set (Appendix). We performed nested PCR twice for all samples, using methods specific for the detection and identification of *H. capsulatum* DNA according to Bialek et al. (*9*). To check for the presence of PCR inhibitors and to avoid false-negative results, we used *H. capsulatum* reference strain G217B as positive control. Sequence analysis detected *H. capsulatum* in 2 of 8 soil samples and in 3 of 9 samples from penguin excreta (Appendix Figure).

We submitted the 5 sequences we detected to GenBank (accession nos. MZ713369–73) and compared them with other *H. capsulatum* sequences. This comparison generated an identity of >98.56% (100% cover) with the deposited sequences of the 100-kDa-like protein gene from *H. capsulatum* and 85% similarity with the sequence of a transcription factor of *Blastomyces dermatitidis* SLH14081 and *B. gilchristii* SLH14081 (GenBank accession nos. XM_045419905 and XM_002628281.2).

**Figure 2:**
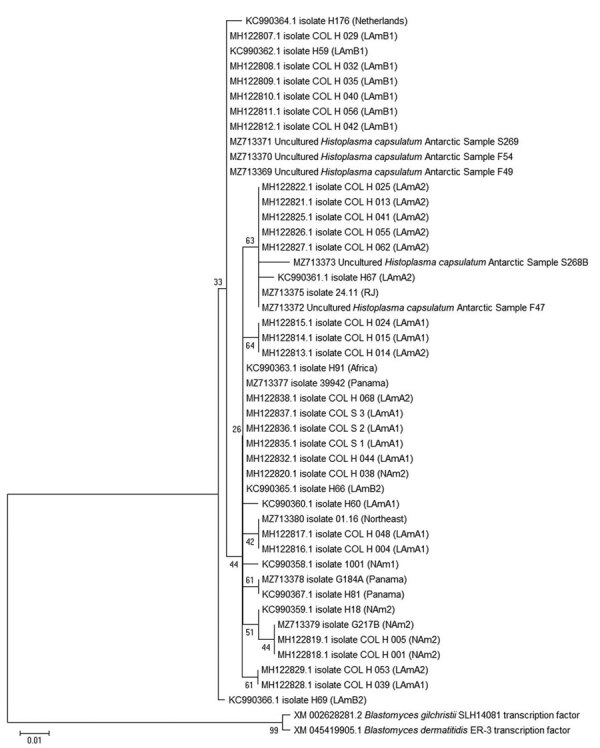
Phylogenetic tree based on the 100-kDa–like protein partial gene sequences of *Histoplasma capsulatum* from Antarctica. The evolutionary history was inferred by using the maximum-likelihood method in in MEGA X software (https://www.megasoftware.net). This analysis involved 46 sequences: 5 from Antarctica samples and 41 representing geographic lineages of *H. capsulatum* in addition to the closest non-*Histoplasma* sequences (*Blastomyces* spp.) downloaded from GenBank (accession numbers shown). The bootstrap percentage of trees in which the associated taxa clustered together is shown next to the branches. Scale bar indicates the number of substitutions per site.

Alignment with GenBank sequences from strains representing the different genetic lineages (Table) demonstrated a difference of up to 14 bp with the 3 haplotypes from Antarctica. The phylogenetic tree formed different groups corresponding to different geographic lineages. Two excreta samples and 1 soil sample grouped with representative strains of the Latin American lineage LAmB1, and 1 soil and 1 excreta sample grouped with a representative strain of LAmA2 lineage. No sample from Antarctica grouped with representative strains of the North America or Panama lineages (Figure 2), indicating a closer association of the newly discovered *Histoplasma* from Antarctica to the South America lineages.

**Table:** List of fungal isolates used in study of *Histoplasma capsulatum* in Antarctica*

Identification	Other names	Source	Location	Phylogenetic species	Accession no.
1001	1001†	Human	Washington, USA	NAm1	KC990358.1
H18	4745‡/1019†/5-1MD	Human	Missouri, USA	NAm2	KC990359.1
H59	2349†/H-0057-I-10	Human	Bogota, Colombia	LAmB1	KC990362.1
H60	2350†/H-0057-I-11	Human	Bogota, Colombia	LAmA1	KC990360.1
H66	2357†/13594/GH	Human	Medellin, Colombia	LAmB2	KC990365.1
H67	2358†/30177/JE	Human	Medellin, Colombia	LAmA2	KC990361.1
H69	2360†/21402/JVM	Human	Medellin, Colombia	LAmB2	KC990366.1
H81	26028‡/2431†	Human	Panamá	Panama	KC990367.1
H91	24295/2444†/8123	Human	Guinea–Liberia border, Africa	Africa/H140 clade	KC990363.1
H176	4741†/CBS 243.69	Human	Netherlands	Netherlands	KC990364.1
COL_S_1	NI	Soil	Colombia	LAmA1	MH122835.1
COL_S_2	NI	Soil	Colombia	LAmA1	MH122836.1
COL_S_3	NI	Soil	Colombia	LAmA1	MH122837.1
COL_H_001	NI	Human	Colombia	NAm2	MH122818.1
COL_H_004	NI	Human	Colombia	LAmA1	MH122816.1
COL_H_005	NI	Human	Colombia	NAm2	MH122819.1
COL_H_013	NI	Human	Colombia	LAmA2	MH122821.1
COL_H_014	NI	Human	Colombia	LAmA2	MH122813.1
COL_H_015	NI	Human	Colombia	LAmA1	MH122814.1
COL_H_024	NI	Human	Colombia	LAmA1	MH122815.1
COL_H_025	NI	Human	Colombia	LAmA2	MH122822.1
COL_H_029	NI	Human	Colombia	LAmB1	MH122807.1
COL_H_032	NI	Human	Colombia	LAmB1	MH122808.1
COL_H_035	NI	Human	Colombia	LAmB1	MH122809.1
COL_H_038	NI	Human	Colombia	NAm2	MH122820.1
COL_H_039	NI	Human	Colombia	LAmA1	MH122828.1
COL_H_040	NI	Human	Colombia	LAmB1	MH122810.1
COL_H_041	NI	Human	Colombia	LAmA2	MH122825.1
COL_H_042	NI	Human	Colombia	LAmB1	MH122812.1
COL_H_044	NI	Human	Colombia	LAmA1	MH122832.1
COL_H_048	NI	Human	Colombia	LAmA1	MH122817.1
COL_H_053	NI	Human	Colombia	LAmA2	MH122829.1
COL_H_055	NI	Human	Colombia	LAmA2	MH122826.1
COL_H_056	NI	Human	Colombia	LAmB1	MH122811.1
COL_H_062	NI	Human	Colombia	LAmA2	MH122827.1
COL_H_068	NI	Human	Colombia	LAmA2	MH122838.1
G184A	H81 lineage	Human	Panamá	Panama	MZ713378.1
G217B	26032‡/1000†/H8	Human	Louisiana, USA	NAm2	MZ713379.1
01.16	INI_01.16	Human	Rio de Janeiro, Brazil	Northeast	MZ713380.1
24.11	IPEC_24.11	Human	Rio de Janeiro, Brazil	RJ	MZ713375.1
39942	NI	Human	Rio de Janeiro, Brazil	Panama	MZ713377.1
S268B	S268	Soil	Potter Peninsula, Antarctic	LAmA2	MZ713373.1
S269	NI	Soil	Potter Peninsula, Antarctic	LAmB1	MZ713371.1
F47	NI	Penguin excreta	Potter Peninsula, Antarctic	LAmA2	MZ713372.1
F49	NI	Penguin excreta	Potter Peninsula, Antarctic	LAmB1	MZ713369.1
F54	NI	Penguin excreta	Potter Peninsula, Antarctic	LAmB1	MZ713370.1

*CBS, Centraalbureau voor Schimmelcultures (Baarn, The Netherlands); INI, Instituto Nacional de Infectologia Evandro Chagas (Rio de Janeiro, Brazil); IPEC, Instituto de Pesquisa Clínica Evandro Chagas (Rio de Janeiro, Brazil); NI, no information. †Roche Molecular Systems Culture Collection, Alameda, CA, USA. ‡American Type Culture Collection, Rockville, MD, USA.

## Conclusions

Moderate temperatures (18°C–28°C), constant humidity (>60%), and a low light environment are thought to characterize suitable ecologic conditions for *H. capsulatum* growth (*10*). Despite the average temperature in Antarctica being below that recognized as ideal for the growth of the fungus, the molecular identification of *H. capsulatum* in 5 of 20 samples collected in Antarctica suggests this species complex could survive at lower temperatures.

Although molecular detection of the fungus does not guarantee its viability, this area of Antarctica is part of an Antarctic Special Protected Area and experiences strong influence of avifauna during the summer period, as well as being host to bird colonies, sea mammal breeding areas, and diverse plant species. Consequently, the soil has high levels of potassium, nitrogen, calcium, and total organic carbon (*11*), which are good conditions for fungal growth. Ideally, *H. capsulatum* should be isolated for complete phenotypic and genotypic study, but it is a slow-growth fungus, and its growth is overtaken by fast-growing fungi. Animal inoculation is often used to recover *H. capsulatum*, but it demands a Biosafety Level 3 facility, which was not available to us.

The molecular detection of *H. capsulatum* in penguin excreta and ornithogenic soil samples leads us to consider the possibility that the fungus could have been imported from outside the continent by migratory birds. Birds are the only terrestrial vertebrates that share with humans the peculiarity of traveling in a few hours across national and intercontinental borders (*12*). During migration, birds have the potential to disperse microorganisms that can be dangerous to humans and a threat to animals (*13*). The fact that high densities of cosmopolitan fungi were found in winter seasonal snow suggests those fungi might be present in air arriving at the Antarctica Peninsula (*14*). Another possibility could be human intervention in the region. Alien microbes, fungi, plants, and animals have arrived over approximately the previous 2 centuries, coinciding with human activity in Antarctica (*6*).

The differentiation of the 7 phylogenetic species in the complex could not be performed with the genetic marker used in this study. However, we detected different haplotypes that grouped with some of those geographically distinct phylogenetic species, suggesting dispersion of the fungus on multiple occasions and, perhaps, indicating adaptation on its way to becoming endemic to the Antarctic Peninsula. The detection of *H. capsulatum* genetically close to representative strains of the Latin American lineages (LAmA2/LAmB1) in Antarctica represents not only the geological history of the continent with South America but the complex dynamics of soil formation and presence of fauna and flora that enable adequate conditions for its maintenance.

This study evaluated a small geographic area of the peninsula, but it has already demonstrated that *H. capsulatum* occurs more broadly than previously suspected (*15*). Considering the capacity of the species to cause life-threatening epidemics and the intensifying human presence on the continent, identifying and monitoring fungi in various Antarctic habitats and animals becomes a fundamental strategy for surveilling emerging systemic mycoses and the flow of these agents between regions, animals, and humans.

Appendix Additional information about molecular detection of *Histoplasma capsulatum* in Antarctica
