# Supplementary material for: Molecular Detection of Histoplasma capsulatum in Antarctica
Source: Emerg Infect Dis. 2022 Oct;28(10):2100–4. doi: 10.3201/eid2810.220046 (PMC9514353; doi:10.3201/eid2810.220046)
Supplement: Appendix — Additional information about molecular detection of Histoplasma capsulatum in Antarctica [file 22-0046-Techapp-s1.pdf]

Article DOI: https://doi.org/10.3201/eid2810.220046

Molecular Detection of *Histoplasma capsulatum* in Antarctica

Appendix

Materials and Methods

Study Area

Environmental samples were collected on the coast of the Potter Peninsula, an Antarctic Specially Protected Area (ASPA N°132) located on King George Island, South Shetland Archipelago. It sits between the Bransfield Strait and the Drake Passage, 62°24′ latitude south, 58°68′ longitude west (Figure 1, https://wwwnc.cdc.gov/EID/article/28/10/22-0046-F1.htm). Winds come mainly from the northwest and west, with gusts that can reach speeds >100 kph. The vegetation in the area is discontinuous and uneven, ranging from nonexistent or sporadic to regions with a predominance of lichens, mosses, or stalk algae. During the summer near the coast, penguins, sea elephants, fur seals, and sea lions can be observed. The material analyzed included 9 samples of penguin excreta, 3 samples fur seal feces, and 8 samples of superficial soil (up to 10 cm deep). The samples were collected during a Brazilian Antarctica expedition in 2020 using sterile material and kept at 2–8°C until transportation to the laboratory at the Oswaldo Cruz Foundation, Rio de Janeiro, Brazil.

Molecular Analysis

The DNA extraction of the environmental material was performed by using the DNeasy PowerSoil kit (QIAGEN, Hilden, Germany) according to manufacturer’s protocol. Crossover contamination was monitored by including 1 sterile water sample at every set of DNA extractions.

Molecular analysis was performed using a nested PCR assay for the detection and identification of *H. capsulatum* DNA by amplification of the gene encoding a 100-kDa-like protein of *H. capsulatum* with specific primers (*9*). The reaction mix was performed in a final volume of 50 μL. Each reaction contained 2.0 μL of template DNA, 1X PCR buffer (Promega,

Madison, USA), 0.2 mM concentration of each deoxynucleoside triphosphate (Cellco, São Carlos, Brazil), 2.0 mM concentration magnesium chloride (Promega, Madison, USA), 1.5 U Go Taq G2 Hot Start Polymerase (Promega, Madison, USA), and 1.0 µM concentration of each primer, HcI (5′-GCG TTC CGA GCC TTC CAC CTC AAC-3′) and HcII (5′-ATG TCC CAT CGG GCG CCG TGT AGT-3′), which amplifies a 391-nt sequence of a 100-kDa-like protein gene of *H. capsulatum*. The reaction for the nested PCR was identical, except the inner primers, which were used instead: HcIII (5′-GAG ATC TAG TCG CGG CCA GGT TCA-3′) and HcIV (5′-AGG AGA GAA CTG TAT CGG TGG CTT G-3′), that amplify a nested PCR product of 210 bp, and 1.0 mM of magnesium chloride. Both amplifications were performed in a SimpliAmp Thermal Cycler (Applied Biosystems, Waltham, USA) with amplification profile including an initial denaturation step at 94°C for 5 min, followed by 35 cycles at 30 s denaturation at 94°C, 30 s annealing at 67°C, 1 min extension at 72°C, and final extension cycle for 5 min at 72°C. Nested PCR was performed twice for all samples. To evaluate the presence of PCR inhibitors and to avoid false-negative results, the samples were run a third time with 0.5ng of genomic DNA of a *H. capsulatum* reference strain G217B, used as positive control. The nested PCR product 210 bp long was sequenced on the automatic capillary Sanger sequencing in an ABI 3730xl-Applied Biosystems machine using the BigDye Terminator v3.1 cycle sequencing kit (ThermoFisher Scientific, USA). Sequencing was performed using both forward and reverse primers. The sequences were manually edited using the software Sequencher 4.10.1 (Gene Codes Corporation, MI, USA), aligned using Muscle algorithm within MEGA X software and blasted against GenBank (https://www.ncbi.nlm.nih.gov/genbank/) to identify the amplified DNA fragment to the species level. Unrooted Maximum Likelihood trees (MEGA X software) were constructed with the sequences from the Antarctic samples and sequences of *H. capsulatum* strains obtained from GenBank, which have been previously characterized by MLST analysis (*3*,*4*). Bootstrap analysis using 1000 replicates was performed to estimate support for the identified clades of the dataset.

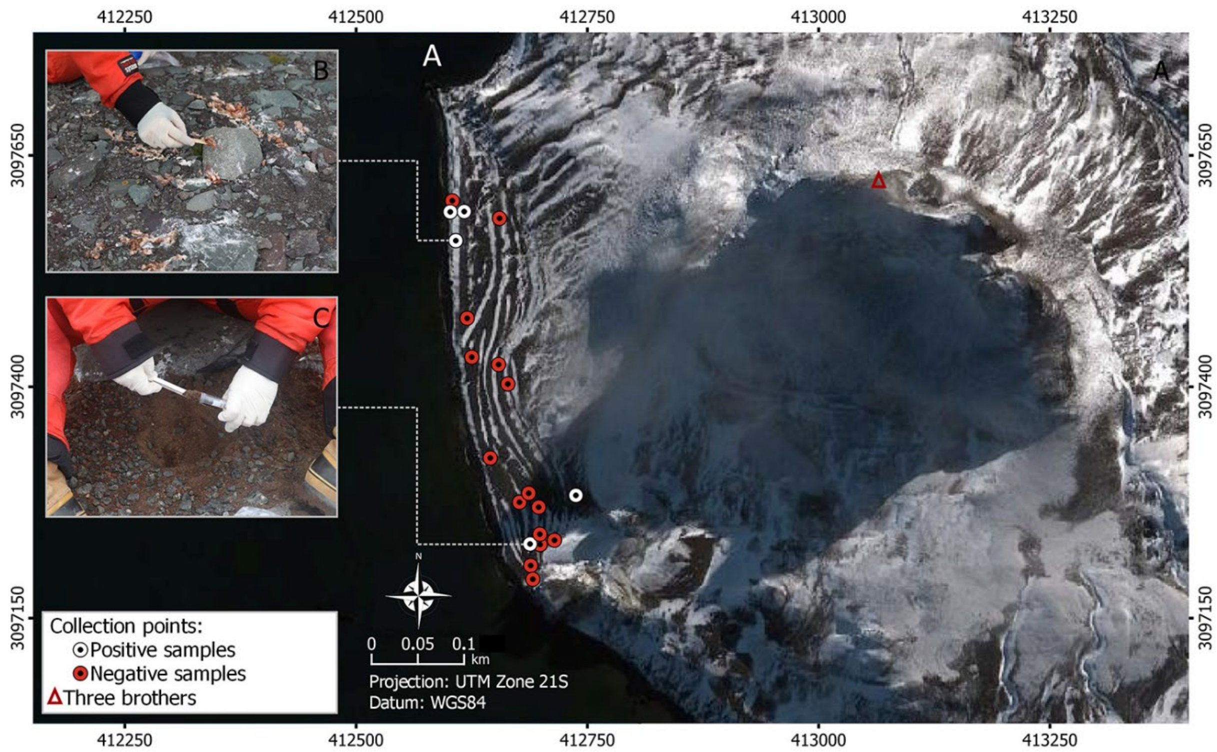

Appendix Figure. Georeferencing of collection points in Antarctic Specially Protected Area nº 132. A) Collection region on the Peninsula Potter; B) Excreta samples collection process; C) Superficial soil samples collection process; Source: Maxar Technologies. Photos (B and C) by Peter Ilicciev.
